# Using Light-Sheet Microscopy to Study Spontaneous Activity in the Developing Lateral-Line System

**DOI:** 10.3389/fcell.2022.819612

**Published:** 2022-04-20

**Authors:** Qiuxiang Zhang, Katie S. Kindt

**Affiliations:** Section on Sensory Cell Development and Function, National Institute on Deafness and Other Communication Disorders, National Institutes of Health, Bethesda, MD, United States

**Keywords:** development, spontaneous activity, hair cell, lateral line efferents, light-sheet microscopy

## Abstract

Hair cells are the sensory receptors in the auditory and vestibular systems of all vertebrates, and in the lateral-line system of aquatic vertebrates. The purpose of this work is to explore the zebrafish lateral-line system as a model to study and understand spontaneous activity *in vivo*. Our work applies genetically encoded calcium indicators along with light-sheet fluorescence microscopy to visualize spontaneous calcium activity in the developing lateral-line system. Consistent with our previous work, we show that spontaneous calcium activity is present in developing lateral-line hair cells. We now show that supporting cells that surround hair cells, and cholinergic efferent terminals that directly contact hair cells are also spontaneously active. Using two-color functional imaging we demonstrate that spontaneous activity in hair cells does not correlate with activity in either supporting cells or cholinergic terminals. We find that during lateral-line development, hair cells autonomously generate spontaneous events. Using localized calcium indicators, we show that within hair cells, spontaneous calcium activity occurs in two distinct domains—the mechanosensory bundle and the presynapse. Further, spontaneous activity in the mechanosensory bundle ultimately drives spontaneous calcium influx at the presynapse. Comprehensively, our results indicate that in developing lateral-line hair cells, autonomously generated spontaneous activity originates with spontaneous mechanosensory events.

## Introduction

Spontaneous activity has been documented in developing sensory systems, including visual, auditory, vestibular and somatosensory systems [reviewed in: (Leighton and Lohmann, 2016)]. Spontaneous activity in sensory cells can play a role locally in synapse maturation and ion channel expression, as well as globally to pattern and refine downstream sensory circuits. For example, in the developing inner ear of mammals, spontaneous activity in sensory hair cells is thought to act locally help establish neuronal connections and globally to shape the tonotopic maps (Tritsch et al., 2007; Ceriani et al., 2019). However, in mammals, hair cells are enclosed in the bony structures of the ear—this location makes it difficult to access and record spontaneous activity *in vivo*. The purpose of this work is to explore the zebrafish lateral line as an *in vivo* model to study spontaneous activity in developing hair cells*.*

The lateral line is composed of superficial clusters of hair cells called neuromasts (Raible and Kruse, 2000). Unlike mammals, the lateral-line system can easily be accessed in intact zebrafish, making, it straightforward to visualize and study developing hair cells *in vivo*. With regard to development, the lateral line forms rapidly—when larvae are 2–3 days old (2–3 days post fertilization (dpf)), the majority of the hair cells are immature. But when larvae are 5–6 days old (5–6 dpf), the majority of the hair cells are mature and the lateral-line system is functional (Kindt et al., 2012; Suli et al., 2012). This rapid developmental trajectory has made zebrafish an excellent system to study hair cell maturation. In addition to rapid development, zebrafish larvae are transparent, making it possible to visualize activity using genetically encoded calcium indicators (GECIs) (Lukasz and Kindt, 2018). The use of GECIs, along with advances in microscopy such as light-sheet fluorescence microcopy (LSFM) has increased the imaging potential of the zebrafish model (Quirin et al., 2016). LSFM uses plane illumination to rapidly image volumes with minimal photobleaching and phototoxicity. Thus, LSFM is a powerful way to image spontaneous activity in zebrafish hair cells, *in vivo*, even over extended periods of time.

In developing hair cells, two forms of spontaneous activity have been documented: 1) coordinated waves of activity among hair cells (Tritsch et al., 2007) and 2) uncoordinated, calcium action potentials that are thought to be generated autonomously (Marcotti et al., 2003; Eckrich et al., 2018). Thus far coordinated waves of activity appear to be a unique feature of the mammalian auditory system. Here, in the developing auditory epithelium, waves of calcium propagate through hair cells and the glia-like supporting cells that surround or are adjacent to hair cells. ATP signaling in supporting cells drives spontaneous activity in nearby auditory hair cells. Independent of these coordinated waves of activity, in mice and zebrafish there are also spontaneous calcium events that are not coordinated among hair cells (Eckrich et al., 2018; Hiu-Tung et al., 2019; Holman et al., 2019). In both mouse auditory hair cells, and zebrafish lateral-line hair cells, Ca_V_1.3 calcium channels present at the hair cell presynapse are required for these events. Although the role of this uncoordinated activity is not fully understood, in the lateral line, we have shown that it can regulate presynapse size in developing hair cells (Hiu-Tung et al., 2019). Currently the origin of these uncoordinated spontaneous events in developing hair cells remains unclear.

Current evidence suggests that spontaneous activity in surrounding supporting cells is not required for uncoordinated spontaneous events in developing hair cells. In addition to surrounding supporting cells, developing hair cells in mouse and zebrafish are contacted by cholinergic efferents that descend from the brainstem (Glowatzki and Fuchs, 2000; Carpaneto Freixas et al., 2021). Furthermore, studies in mice have shown that these efferents can modulate hair cell activity during development (Glowatzki and Fuchs, 2000; Katz et al., 2004; Holman et al., 2019). Further, when the α9/α10 acetylcholine receptors required for this cholinergic modulation are disrupted, the patterns of hair cell spontaneous activity in the auditory epithelium of mice are altered (Clause et al., 2014). But whether these descending efferents are spontaneously active in hair cell systems and whether they can trigger spontaneous activity in developing hair cells is not known.

Our study applies LSFM to study spontaneous calcium activity *in vivo*, in the zebrafish lateral-line system. We show that spontaneous calcium activities are present in several cell types in the periphery of the developing lateral line: hair cells, supporting cells, as well as cholinergic efferents that innervate hair cells. Using two-color functional imaging, along with pharmacology, we find that hair cell spontaneous activity occurs independent of activity in supporting cells and cholinergic efferents. Instead, developing hair cells autonomously generate spontaneous events. By using a membrane-localized calcium indicator in hair cells, we show that spontaneous calcium activity occurs in two distinct domains: the mechanosensory bundle and the presynaptic compartment. Further, our genetic and pharmacological analyses reveal that in lateral-line hair cells, spontaneous mechanosensory activity in the mechanosensory bundle drives spontaneous calcium influx at the presynapse. Thus, mechanosensory activity is the main source of spontaneous activity in lateral-line hair cells.

## Methods

### Zebrafish Husbandry and Strains

Zebrafish (Danio rerio) were raised at 28°C on a 14:10 h light/dark cycle. Larvae at 2–6 days post fertilization (dpf) were used for the experiments and were maintained in E3 embryo medium (in mM: 5 NaCl, .17 KCl, 0.33 CaCl_2_, and 0.33 MgSO_4_, buffered in HEPES pH 7.2) at a constant temperature of 28°C in an incubator. Because sex is not yet determined at these ages, we did not consider the animal’s gender in our research. Zebrafish work performed at the National Institute of Health was approved by the Animal Use Committee under animal study protocol #1362-13. The following previously established mutant and transgenic zebrafish strains were used in this study: *Tg(myo6b:memGCaMP6s)* ^*idc1*^ (Jiang et al., 2017), *Tg(myo6b:RGECO1)* ^*vo10*^ (Maeda et al., 2014), *Tg(UAS:GCaMP6s)* ^*mpn101*^ and *Tg(chat:Gal4)* ^*mpn202*^ (Förster et al., 2017), *pcdh15a* ^*th263*^ *(R306X)* (Seiler et al., 2005), and *cav1.3a/cacna1d* ^*tn004*^ *(R284C)* (Sidi et al., 2004).

### Vector Construction and Creation of New Transgenic Lines

We generated two additional stable transgenic fish lines for this study. Plasmid construction to create these lines was based on the tol2/gateway zebrafish kit (Kwan et al., 2007)*.* Plasmid DNA and tol2 transposase mRNA were injected into zebrafish embryos as previously described (Kwan et al., 2007). Using this approach, in addition to the previously established transgenic lines listed above, the transgenic lines *Tg(she:GCaMP6s)* ^*idc17*^ and *Tg(myo6b:GCaMP6s)* ^*idc18*^ were created for and used in this study. These lines used the supporting cell-specific promoter (*she*) (Quillien et al., 2017) or hair cell-specific promoter (*myo6b*) (Obholzer et al., 2008) to express GCaMP6s in order to image spontaneous calcium activity in the cytosol.

### Sample Preparation for 4D Spontaneous Calcium Imaging *In Vivo*

Larvae at 2–6 dpf were first anesthetized with 0.03% Ethyl 3-aminobenzoate methane sulfonate salt (Sigma-Aldrich, St. Louis, MO, United States) and pinned onto a Sylgard-filled recording chamber (I-2450, ASI, Eugene, OR, United States). After pinning, 125 μM α-Bungarotoxin (Tocris, Bristol, United Kingdom) was injected into the heart of intact larvae to suppress movement. Larvae were then rinsed with extracellular imaging solution (in mM: 140 NaCl, 2 KCl, 2 CaCl_2_, 1 MgCl_2,_ and 10 HEPES, pH 7.3) and allowed to recover. To image the spontaneous calcium activity, the larvae were kept in extracellular imaging solution without applying any external stimuli.

### Pharmacology

All drugs were prepared in extracellular solution with 0.1% DMSO (except no DMSO was used with BAPTA). Animals were bathed in each drug (except BAPTA) for at least 15 min prior to imaging. For BAPTA treatment, animals were incubated in BAPTA for 15 min, followed by a wash with extracellular solution. Listed in the table above are the concentrations of the drugs used in this study.

**Table:** 

Drug	Mode of action	Vendor	Working concentration
FFA	Gap junction blocker antagonist	Sigma-Aldrich	25 μM
MRS2500	P2yr1 selective antagonist	Tocris	1 μM
Thapsigargin	Potent inhibitor of sarco/endoplasmic reticulum Ca^2+^-ATPases (SERCA)	Sigma-Aldrich	250 nm
α-bungarotoxin (α-Btx)	Potent inhibitor of α9 or α10 nicotinic acetylcholine receptor (nAChR)	Tocris	10 μM
Apamin	SK channel blocker	Tocris	10 μM
Isradipine	L-type Ca2+ channel CaV1.3a antagonist	Sigma-Aldrich	10 μM
BAPTA-tetrasodium salt	Disrupts hair-bundle tip links	Thermofisher	5 mM

### Light-Sheet System Construction

4D spontaneous calcium activity of zebrafish neuromast was imaged by a homebuilt dual-view inverted selective-plane illumination microscope (diSPIM). The microscope was built and aligned according to the previously described protocols (Kumar et al., 2014). Briefly, the bulk of the optomechanical hardware, automated stages, laser scanners, piezo elements for focus control, and control electronics were purchased from Applied Scientific Instruments (ASI, Eugene, OR, United States). Each arm of the diSPIM (SPIMA and SPIMB) consists of a water-dipping objective (Nikon CFI NIR Apo 40X Water DIC N2, Nikon, Melville, NY, United States) either emitting the laser light for excitation or collecting signals from the sample using an ORCA-Flash 4.0 sCMOS camera (Hamamatsu Photonics, Hamamatsu City, Shizuoka, Japan). Two optically pumped semiconductor laser (OBIS 488 nm LX 30 mW and OBIS 561 nm LS 80 mW, Coherent, CA, United States) were combined with a beam combiner (OBIS Galaxy, Coherent, CA, United States) and then routed to a fiber optic switch (eol 1x2 VIS, LEONI, VA, United States) for output laser wavelength selection. Two outputs from the fiber optic switch were fiber-coupled to two diSPIM head laser scanners. The open-source platform Micro-Manager (https://micro-manager.org/) (Edelstein et al., 2010) was used for hardware interfacing, data capture, and storage. A TMC vibration isolation lab table (63P-9012M, TMC, Boston, MA, United States) was used to house the light-sheet system to minimize disturbance from external movement or vibration.

### DiSPIM Fast Acquisition of Volumes to Detect Spontaneous Calcium Signals

**FIGURE 1:**
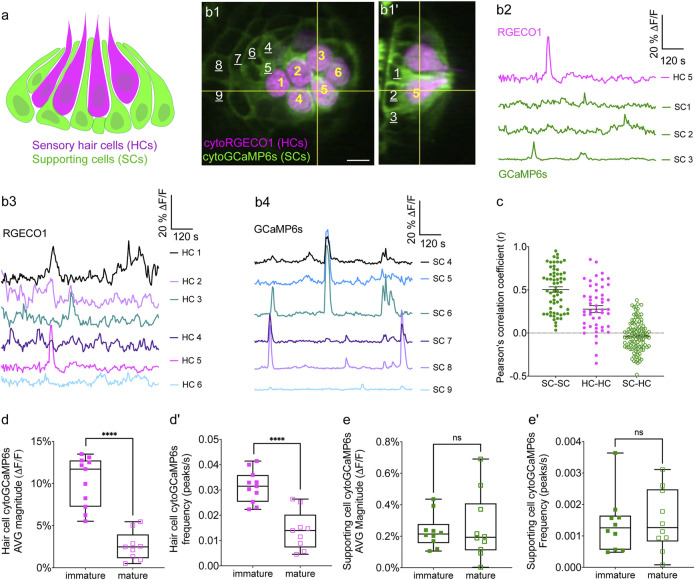
Simultaneous imaging of spontaneous activities in hair cells and supporting cells. **(A)** Cartoon of a neuromast organ illustrating sensory hair cells (magenta) and the surrounding supporting cells (green). **(B1–B1′)** High-resolution images taken using two arms of our diSPIM system show a representative, day 3, double transgenic neuromast expressing cytoRGECO1 (hair cells, numbered in yellow) and cytoGCaMP6s (supporting cells, a subset is numbered in white, underscored). A top-down view **(B1)** and the corresponding side view of its orthogonal projection **(B1′)** clearly show the hair cells and the surrounding supporting cells at day 3. **(B2)** Temporal curves of spontaneous calcium activity in hair cell 5 (labeled in **B1–B1′**) and its three surrounding supporting cells (labeled in **B1**′) within the 15 min recording window show distinct time courses. **(B3)** Temporal curves of spontaneous calcium activity in the cluster of six hair cells (labeled in **B1**) reveals distinct response profiles. **(B4)** Temporal curves of spontaneous calcium activity in the seven supporting cells (labeled in **B1**) reveals some correlation and synchronization among neighboring supporting cells. **(C)** Comparisons of Pearson’s R of spontaneous calcium activities at day 3 in neighboring supporting cells, neighboring hair cells and hair cells with its surrounding hair cells, *n* = 6 neuromasts for HC-HC or SC-SC R values and *n* = 4 neuromasts for HC-SC R values. **(D–D′)** Box plots showing quantification of the average magnitude **(D)** and frequency **(D′)** of spontaneous calcium activity in immature (day 3) and mature (day 6) hair cells. **(E–E′)** Box plots showing quantification of the average magnitude **(E)** and frequency **(E′)** of spontaneous calcium activity in supporting cells in mature (day 6) and immature (day 3) neuromasts. Each dot in **(D–E′)** represents one neuromast. An unpaired *t*-test was used in **(D–E′)**. ****p < .0001. Scale bar = 5 μm.

**FIGURE 2:**
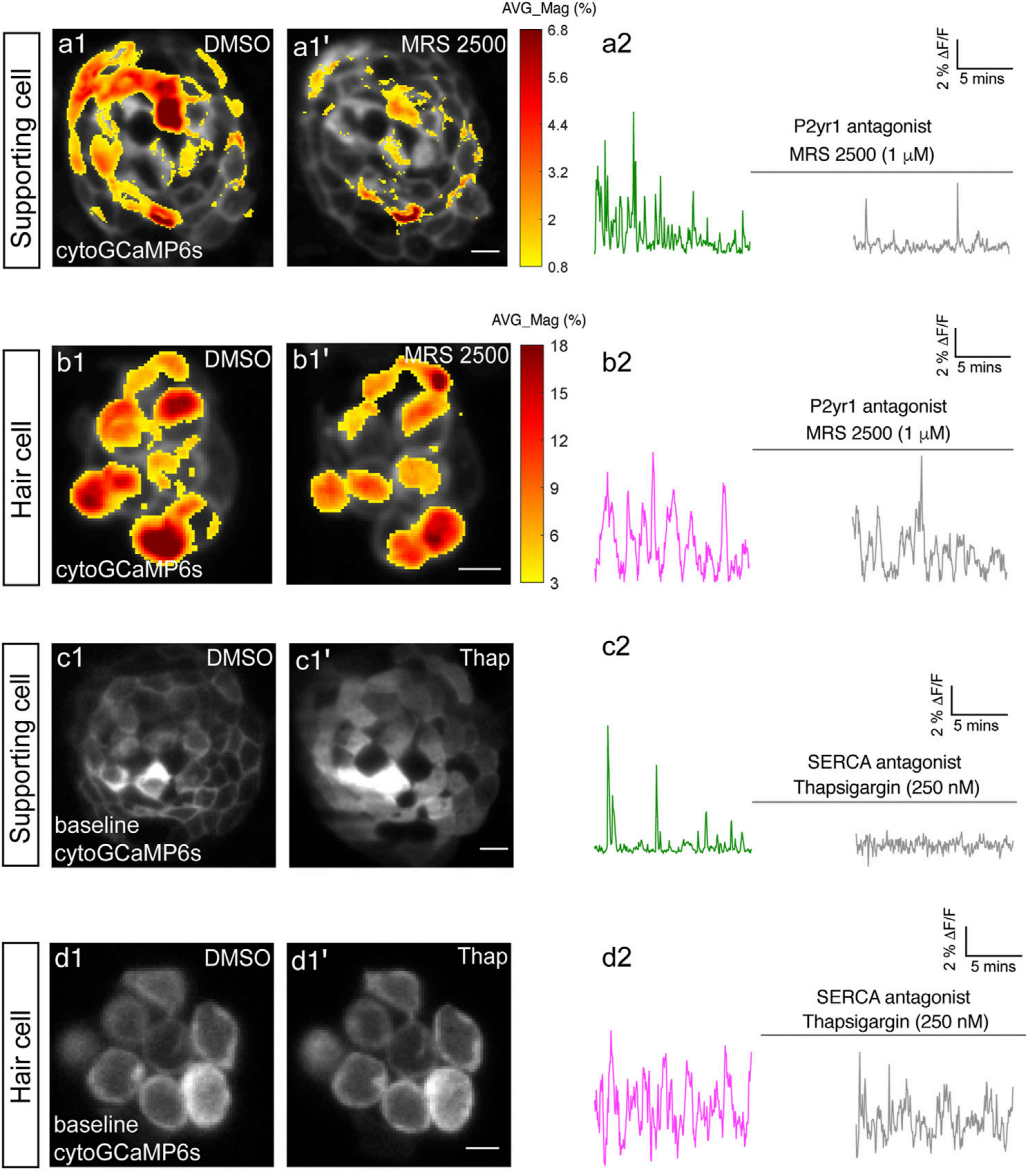
P2yr1 signaling is required for spontaneous activity in supporting cells but not immature hair cells. The spatial patterns of the mean spontaneous calcium activities of the supporting cells **(A1–A1′)** or hair cells **(B1–B1′)** in DMSO and after 15 min of treatment with 1 µM MRS2500. Measurements were performed in immature neuromasts at day 3. The ΔF/F GCaMP6s signals were averaged over each 900 s interval (pre- and post-treatment) and then colorized according to the heat map and superimposed onto a baseline image. The corresponding temporal curves of the mean signal magnitude across the whole neuromast in supporting cells **(A2)** and in hair cells **(B2)** in DMSO and after 15 min of treatment with 1 µM MRS2500. The cytosolic baseline calcium in the supporting cells **(C1–C1′)** and hair cells **(D1–D1′)** in DMSO and after 15 min of treatment with 250 nM Thapsigargin. Measurements were performed in immature neuromasts at day 3. The corresponding temporal curves of the mean signal magnitude across the whole neuromast in supporting cells **(C2)** and in hair cells **(D2)** in DMSO and after 15 min of treatment with 250 nM Thapsigargin. Scale bar = 5 μm.

**FIGURE 3:**
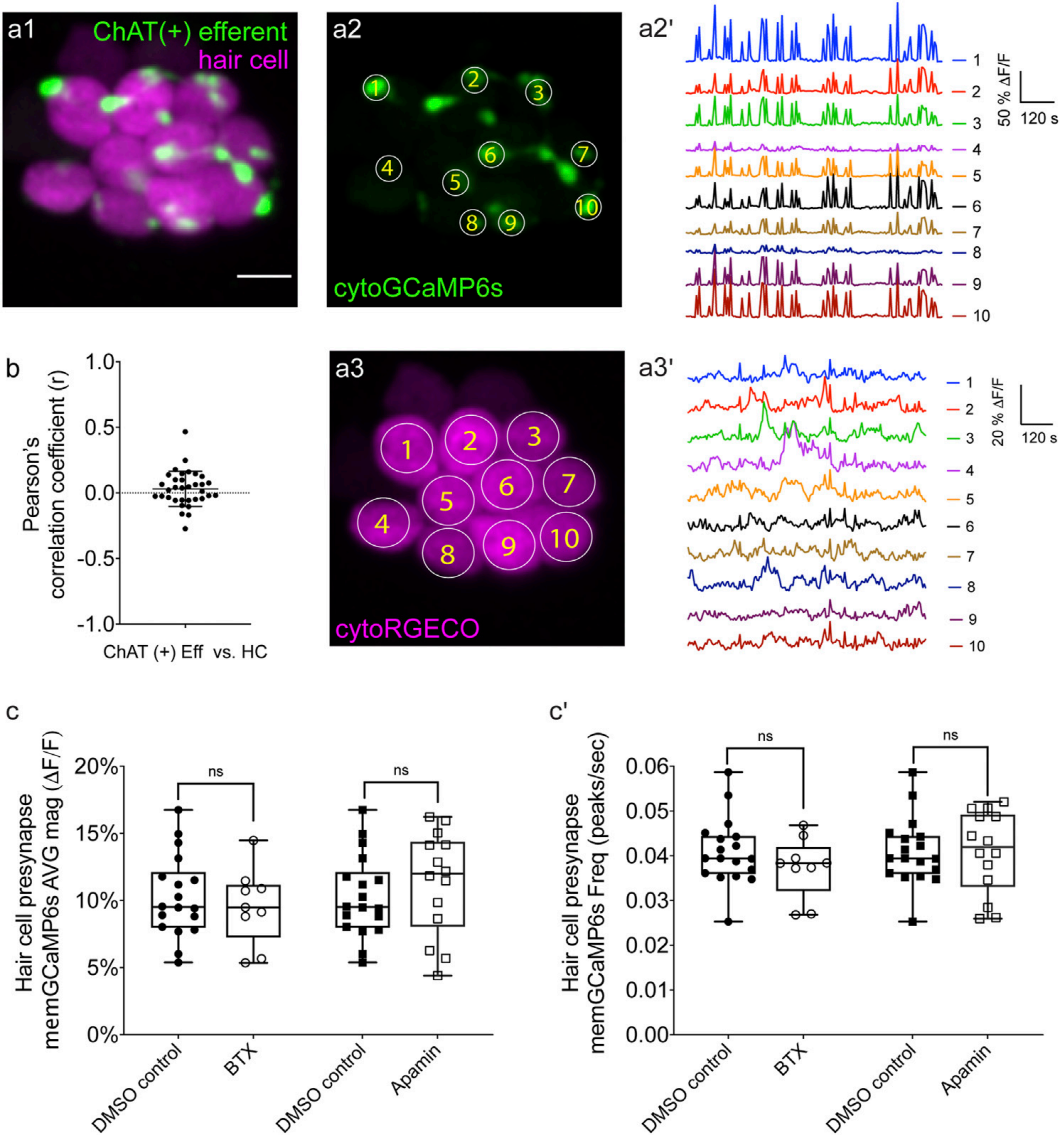
Two-color imaging of spontaneous activities in hair cells and cholinergic efferent terminals. **(A1)** A representative Max-projection image of a double-transgenic zebrafish line expressing the red GECI cytoRGECO1 in hair cells and the green GECI cytoGCaMP6s in the cholinergic efferents terminals at day 3. **(A2)** Image depicting cholinergic efferent terminals with individual terminals contacting different hair cells in **(A1)** are labeled accordingly. **(A2′)** The corresponding temporal curves of spontaneous calcium activities of the 10 efferent terminals indicated in **(A2)**. **(A3)** Image of the individual hair cells in a1 labeled according to innervating efferent terminal. **(A3′)** The corresponding temporal curves of spontaneous calcium activities of the 10 hair cells indicated in **(A3)**. **(B)** Pearson’s R values of spontaneous calcium activity between each hair cell and its contacting cholinergic efferent terminal, *n* = 34 hair cell-efferent terminal pairs from four neuromasts at day 3. Box plots showing the average magnitude **(C)** and frequency **(C')** of spontaneous calcium activity at the hair cell presynapse in DMSO and after the treatment with 10 μM α-Btx and 10 μM apamin. Each point in **(C–C′)** represents one neuromast. All measurements were performed in immature neuromasts at day 3. A paired *t*-test was used in **(C–C′)**. Scale bar = 5 μm.

For fast, single wavelength imaging of spontaneous cytoGCaMP6s or memGCaMP6s calcium signals, one arm of the diSPIM (SPIMA) was used to acquire volumes at a rate of ∼0.33 Hz (every 3 s) for 15 min. 10 (efferent) or 20 (hair cell and supporting cell) slices were acquired per volume (1 μm spacing) at 10 ms and 256 × 256 pixels per slice with 2 × 2 binning (325 nm per pixel with 2 × 2 binning). This volume encompasses the entire neuromast. Each volume was acquired in 100 (efferent) or 200 ms (hair cell or supporting cell); this acquisition speed was sufficient to measure spontaneous GCaMP6s-dependent signals near simultaneously within the volume. Further, this volumetric acquisition speed (100 or 200 ms), volumetric frame rate (every 3 s) and resolution were optimal to record spontaneous events without any significant photobleaching. No additional activity was observed with a faster acquisition speed or volumetric frame rate, or with a higher resolution. This fast, single wavelength imaging was used to acquire cytoGCaMP6s signals in either hair cells or supporting cells (Figures 1D–E; Supplementary Figure S1; Figure 2; Supplementary Figures S3, S5) or to measure cytoGCaMP6s signals in efferent terminals (Supplementary Figures S6B, C–C′). In addition, fast, single wavelength imaging was also used to simultaneously acquire memGCaMP6s signals in neuromast hair bundles and presynapses (Figures 3C–C′, 5, 6).

Fast, two-color imaging was performed using a similar approach. One arm of the diSPIM (SPIMA) was used to sequentially acquire volumes of green and red GECI signals at rate of ∼0.2 Hz (every 5 s) for 15 min. Eight slices were acquired per volume (2 μm spacing) at 5 ms and 256 × 256 pixels per slice with 2 × 2 binning. Each volume was acquired sequentially (GCaMP6s volume followed by RGECO1 volume) with an acquisition speed of 242 ms for the 2 volumes (GCaMP6s and RGECO1). These calcium imaging parameters were optimized to minimize noise and ensure reliable correlation values. This acquisition speed and spacing was sufficient to measure spontaneous GCaMP6s- and RGECO1-dependent signals near simultaneously within the volumes and optimal to prevent RGECO1 photobleaching. This approach was to image calcium signals in hair cells (cytoRGECO1) along with calcium signals in supporting cells (cytoGCaMP6s) or efferent terminals (cytoGCaMP6s) (Figures 1B2–C; Supplementary Figure S2; Figures 3A1–A3′; Supplementary Figures S6A–A3′). For quantitative measurements of spontaneous calcium signals, these fast, single wavelength or two-color 3D time-series were processed and used for analyses.

### DiSPIM Acquisition of High-Resolution Volumes for Spatial Delineation of Morphology

To clearly delineate neighboring cells and cell-adjacent synaptic arbors in our fast acquisitions, and create 4D movies (Supplementary Movie S1), we acquired additional high-resolution reference image stacks. For this slower, higher resolution acquisition, volumes were acquired from each of the two views (SPIMA and SPIMB) with 166 slices (0.5 μm spacing), at 512 × 512 pixels per slice and 1 × 1 binning (162.5 nm per pixel). After high-resolution imaging, images were cropped, and the backgrounds were subtracted in ImageJ (Schneider et al., 2012). The two image stacks from SPIMA and SPIMB views were then co-registered and deconvolved jointly to obtain a single volumetric image stack with isotropic spatial resolution and an isotropic voxel spacing (0.1625 × 0.1625 × 0.1625 μm/pixel). Custom software built in C++/CUDA (Guo et al., 2020) was used to conduct image registration and deconvolution on a graphics processing unit (GPU) card. The image registration process started with transforming the image stack from SPIMB with rotation, translation, or scaling and then overlaid and compared with those from SPIMA. By minimizing a cost function via Powell’s method (http://mathfaculty.fullerton.edu/mathews/n2003/PowellMethodMod.html), the best transformation matrix was obtained for registration. The images were then deconvolved jointly by an “unmatched back projector” method, which could significantly accelerate deconvolution (Guo et al., 2020). The updated code published by Min Guo and Hari Shroff can be download from GitHub at: (https://github.com/eguomin/regDeconProject; https://github.com/eguomin/diSPIMFusion; https://github.com/eguomin/microImageLib).

We also characterized the actual resolution of our diSPIM system using carboxylate-modified fluorescent beads (0.1 µm, F8803, Thermo Fisher Scientific, MA, United States), diluted 100 times before use. The full width at half-maximum (FWHM) numbers were calculated for eight beads along all axes before and after joint deconvolution (Kumar et al., 2014). Before deconvolution, the FWHM of a bead was close to 0.5 μm (lateral) and 1.5 μm (axial). After registration of the two views (SPIMA and SPIMB) and joint deconvolution, the bead FWHM was approximately isotropic with 0.32 μm ± 0.04 std (lateral) and 0.41 μm ± 0.05 std (axial) resolution.

### Quantification of Spontaneous Calcium Signals

3D times-series imaged at fast acquisition were used to quantify spontaneous calcium signals. The stacks first registered by the application of the imageJ macro, Correct 3D Drift (Arganda-Carreras et al., 2006; Parslow et al., 2014). Image stacks with excessive X-Y drift or drift in Z were discarded from our analyses. After drift correction, volumes were converted into 2D time-series images by using the ImageJ Maximum Intensity Z-projection function. Volumes were Max-projected as follows, single wavelength: hair cell memGCaMP6s and cytoGCaMP6s (every 6 µm), supporting cell cytoGCaMP6s (every 4 µm), efferent cytoGCaMP6s (entire 10 µm volume), two-color: hair cell cytoRGECO (every 8 µm), supporting cell cytoGCaMP6s (every 4 µm), efferent cytoGCaMP6s (entire 16 µm volume). These projections reduced signal to noise without an overlap in signal between neighboring cells or terminals. After Max-projection another ImageJ macro, StackReg (Thevenaz et al., 1998) was used to further correct movement artifacts if necessary. If stacks could not be adequately corrected for X-Y drift they were discarded from analyses. For whole neuromast quantification, projections containing the majoring of cells or terminals were selected for analyses. For Pearson’s correlation analyses, projections centered the cell or terminal of interest were used.

For quantification, a circular ROI was placed on each cell or terminal (diameter of ROI: ∼5 µm hair cell base; ∼2 µm hair bundle or efferent terminal; ∼3 µm supporting cell soma). Using these ROIs, the fluorescent intensity value within each ROI was obtained for each time point in each cell or terminal. Next the fluorescent intensity values (∆F) within each ROI were plotted for further processing in MATLAB R2020a (Mathworks, Natick, MA, United States). The baseline (F_0_) was calculated for each timepoint using the MATLAB imerode function to create a plot of ∆F/F_0_ values. Using these ∆F/F_0_ values we quantified 1) Pearson’s correlation coefficients, 2) the magnitude, frequency and duration of individual spontaneous peaks in GCaMP6s recordings and 3) the average magnitude of spontaneous signals above baseline during the recording period.

To calculate Pearson’s correlation coefficient (R), Prism 8 (Graphpad, San Diego, CA, United States) was used. The correlation between two (∆F/F_0_) measurements was computed at all time points during the 15 min recording period. This approach was used to correlate activity within a hair cell (hair bundle and presynapse) and between a hair cell and the innervating efferent terminal. To calculate a Pearson’s correlation coefficient between a cell and its 3–4 neighboring cells, a correlation was calculated for each neighbor, and all correlations were averaged to generate one Pearson’s correlation coefficient value per cell. For comparisons of efferent terminals within a neuromast, a Pearson’s correlation coefficient was calculated for all terminal pairings.

We detected peaks in our ∆F/F_0_ GCaMP6s traces in MATLAB using the built-in peak detection function findpeaks. After peak detection we calculated the average magnitude, frequency and duration of peaks for each ROI over the whole recording period (15 min). Then we averaged this information per neuromast to create a single value for each neuromast.

To calculate average magnitude of spontaneous GCaMP6s signals, all values of ∆F/F_0_ less than 10% in the recording were removed. Values below 10% were considered noise; therefore, signals above 10% ∆F/F_0_ were our threshold value for a true GCaMP6s signal. At the hair cell presynapse a 10% threshold was confirmed by imaging spontaneous memGCaMP6s signals in the presence of isradipine where no signals were observed (Figure 5). The average magnitude of spontaneous activity per cell or terminal was obtained by dividing the integral or sum of GCaMP6s signals (∆F/F_0_ > 10%, our threshold value) during the whole recording period by the total frames. The average magnitude was calculated for all hair bundles, presynapses, hair cells, supporting cells and efferent terminals within a neuromast. These values were then averaged to create a single value for each neuromast.

### Spatial and Temporal Visualization GCaMP6s Signals in Image Stacks

To better observe the spontaneous calcium signals temporally and spatially (Figure 2, Supplementary Figure S4), fluorescence images were scaled and represented using color maps, with red indicating an increase in calcium signal relative to the resting period (baseline). The procedure to obtain these color maps has been described previously (Lukasz and Kindt, 2018).

In Supplementary Figure S4, spatial and temporal changes in spontaneous activity in a single supporting cell was obtained by subtracting each image from baseline (reference image just prior to the rise signal) to represent the relative change in fluorescent signal (∆F). Using this method, the spatial changes in ∆F (via the heat map) were visualized at multiple time points during a spontaneous event.

In Figure 2, the spontaneous activity in supporting cells and hair cells was represented spatially by computing the average magnitude (∆F/F_0_) over the whole recording period (15 min) pixel by pixel. The ∆F/F_0_ values were then converted into a heat map and overlaid on a single grayscale morphological image. The accompanying cytoGCaMP6s traces in Figure 2 were obtained by placing an ROI over the entire neuromast during the pre- and post-drug incubation time windows.

### Statistical Analysis

All data were analyzed and plotted with Prism 8. Values in the text and data with error bars on graphs and in text are expressed as mean ± SEM. All experiments were performed on a minimum of three animals. From these three animals, at least four neuromasts were examined. All experiments were repeated on two independent days. These numbers were adequate to provide statistical power to avoid both Type I and Type II error. Datasets were confirmed for normality using a D’Agostino-Pearson omnibus normality test. Statistical significance between two conditions was determined by either an unpaired or paired t-test, or a Kruskal–Wallis test as appropriate. Prism 8 was used to create correlation heat maps.

## Results

### Fast, Volumetric, *In Vivo* Imaging of Spontaneous Calcium Activity in Developing Hair Cells

Our previous work demonstrated that spontaneous presynaptic calcium activity in developing lateral-line hair cells is important for proper presynapse formation (Hiu-Tung et al., 2019), yet how presynaptic calcium influx was initiated remained unclear. To study spontaneous activity during development in more detail, we constructed a dual-view inverted selective plane illumination microscope (diSPIM) (Kumar et al., 2014). This light-sheet microscope is specialized for fast, continuous imaging of volumes over long-time windows, with minimal photodamage or bleaching. In addition, this microscope can acquire high-resolution volumes with isotropic resolution in XYZ.

We tested the capability of this fluorescence microscope to detect spontaneous calcium activities in transgenic zebrafish lines expressing the cytosolic GECIs cytoGCaMP6s or cytoRGECO1 in hair cells. Using this approach, we observed robust spontaneous calcium signals in immature hair cells (day 3; Figures 1B1–B3, D–D′; Supplementary Movies S1, S2). Consistent with our previous results where we used a membrane-localized indicator, memGCaMP6s to monitor spontaneous presynaptic calcium activity, cytoGCaMP6s signals were dramatically reduced when hair cells matured (day 6; Figures 1D–D′). We found that this microscope system was suitably fast for imaging the time course of these calcium signals in volumes (83 μm × 83 μm × 20 µm volumes acquired within 100 ms every 3 s) that encompass the entire neuromast organ. Despite continuous, volumetric imaging over long-time windows (15 min), we observed minimal-to-no photodamage or bleaching (Figure 1B3). Overall, our data indicates that the diSPIM microscope is well-suited to rapidly measure spontaneous calcium activities within entire neuromast organs.

### Spontaneous Calcium Activity is Present in Zebrafish Supporting Cells

Similar to the mammalian inner ear, hair cells in the zebrafish lateral line are isolated and surrounded by supporting cells (Figures 1A–B′). Furthermore, studies in the developing inner ear in mice have shown that spontaneous activity in present in hair cells and supporting cells. Therefore, we tested whether zebrafish supporting cells were spontaneously active during development. To measure spontaneous calcium activity, we created a transgenic zebrafish line expressing the cytosolic GECI cytoGCaMP6s in supporting cells (Figures 1B1–B1′). Using this line, we observed that in developing neuromasts, spontaneous calcium signals were also present in supporting cells (day 3; Figures 1B2, B4, E–E′; Supplementary Movie S3).

We compared the properties of the spontaneous signals detected in supporting cells with those detected in the hair cells. For our comparison we used transgenic lines that expressed same GECI, cytoGCaMP6s in either supporting cells or hair cells (Supplementary Figure S1). Our analyses revealed that the average magnitude of cytoGCaMP6s signal above our baseline threshold during the 15-min recording window (average magnitude; Supplementary Figure S1A) was much higher in hair cells than that in supporting cells. Furthermore, there were also significantly more spontaneous calcium events occurring in the hair cells compared with supporting cells (peaks per second; Supplementary Figure S1A′). In addition to more spontaneous calcium events, each spontaneous calcium event lasted much longer in the hair cells compared to supporting cells (duration, Hair cells: 14.22 s ± 0.68, Supporting cells: 9.06 s ± 0.51; Supplementary Figure S1A′′). Despite more events with a longer time course, on average, the peak magnitudes were comparable between hair cells and supporting cells (average peak mean; Supplementary Figure S1A′′′). These data indicate that in the zebrafish lateral-line system, spontaneous calcium activity is present in both hair cells and supporting cells.

Our work indicates that spontaneous calcium signals in hair cells are restricted to development (Figures 1D–D′). Interestingly, we found no significant difference in spontaneous cytoGCaMP6s signals in supporting cells when we compared immature (day 3) to mature (day 6) neuromast organs (Figures 1E–E′). Overall, our work indicates that spontaneous activities are present in both hair cells and supporting cells in the zebrafish lateral line. Although hair cell spontaneous activity occurs primarily during development (Figures 1D–D′), in supporting cells, this activity is retained upon sensory system maturation.

### Spontaneous Calcium Activities in Hair Cells and Supporting Cells Do Not Coincide

To understand if spontaneous calcium activities in hair cells and supporting cells are linked, we performed two-color (red and green) calcium imaging. Here we simultaneously imaged the activity in both hair cells and supporting cells, using a double-transgenic zebrafish line expressing the red cytosolic GECI cytoRGECO1 in hair cells along with the green cytosolic GECI cytoGCaMP6s in supporting cells (Figures 1B1–B1′). We focused our analyses on immature neuromasts (day 3) when spontaneous calcium activity is robust in hair cells. Using our diSPIM microscope, along with these calcium reporters, we were able to simultaneously detect spontaneous calcium activity in all hair cells and supporting cells within whole neuromast organs (Figures 1B,C; Supplementary Figure S2; Supplementary Movie S4).

Next, we investigated whether there was a relationship between the spontaneous calcium activities in hair cells and the surrounding supporting cells during development (day 3). In addition to the fast-volumetric calcium imaging, diSPIM light-sheet systems can also acquire high-resolution, 3D image volumes that can be viewed in a top-down view (Figure 1B1) or a corresponding side view (Figure 1B1′) with comparable resolution (Kumar et al., 2014). These isotropic 3D volumes enabled us to spatially delineate all hair cells, as well as all surrounding supporting cells (Figures 1B1,B1′; Supplementary Figures S2A1–A1′, B1–B1′, C1–C1′). Using these isotropic volumes to delineate cells, we observed that the spontaneous calcium activities in hair cells and neighboring supporting cells (quantified from our fast-volumetric calcium imaging) did not coincide. For example, the side-view image in Figure 1B1′ clearly highlights a hair cell (HC 5) and its three surrounding supporting cells (SC 1–3). By plotting the temporal curves of these four cells (Figure 1B2), we found that both hair cells and the supporting cells were spontaneously active. But the temporal response profile in the hair cell was distinct from its surrounding supporting cells (Supplementary Figures S2C1–C3; Pearson’s *R* = 0.19, 0.19 and −0.01). Similar results were obtained for other hair cell and supporting-cell pairings in this example (Supplementary Figures S2A1–B3). We extended this analysis and calculated the Pearson’s correlation coefficients from cell pairings across many developing neuromasts organs (day 3) and overall found no correlation in spontaneous calcium activities between hair cells and surrounding supporting cells (Figure 1C, Pearson’s *R* = −0.04 ± 0.02, *n* = 4 neuromasts). In summary, analysis of our two-color functional imaging in hair cells and supporting cells revealed that there was little to no correlation in spontaneous calcium activities between immature hair cells and surrounding supporting cells.

After determining that there was no correlation in spontaneous calcium activities between hair cells and supporting cells, we examined whether there was a correlation among populations of hair cells or supporting cells within neuromast organs. Within a given neuromast, we observed that each hair cell showed distinct response profiles (example, Figure 1B3) and the correlation between any two neighboring hair cells was low (example, Supplementary Figures S2D1–D2). When we examined spontaneous calcium activity between neighboring hair cells across many developing neuromast organs, quantification revealed little to no correlation (Figure 1C, Pearson’s *R* = 0.28 ± 0.04, *n* = 6 neuromasts). In contrast to hair cells, by plotting temporal curves of spontaneous calcium activity from individual supporting cells, we observed clear examples of synchronized activities (examples, Figure 1B4; Supplementary Figures S2D1,D3). When examining the spontaneous calcium activity between neighboring supporting cells, quantification revealed a relatively high correlation (Figure 1C, Pearson’s *R* = 0.50 ± 0.03, *n* = 6 neuromasts). Numerous studies have demonstrated that supporting cells in mammals and zebrafish are electrically coupled via gap junction channels (Kikuchi et al., 2000; Zhang et al., 2018). Therefore, one possible reason for synchronized activities between supporting cells are these gap junction channels. Consistent with this idea, we found that blockage of gap junctions with flufenamic acid (FFA) significantly reduced the correlation in spontaneous calcium activity between neighboring supporting cells (control: Pearson’s *R* = 0.45 ± 0.03; 10 µM FFA: Pearson’s *R* = 0.08 ± 0.02, *n* = 4 neuromasts).

Together, our results suggest that there is no obvious temporal relationship between spontaneous calcium activities in hair cells and supporting cells. Furthermore, distinct temporal properties indicate the source or mechanism underlying these two distinct spontaneous calcium signals may also be different.

### P2yr1-ER Signaling Underlies Spontaneous Calcium Activity in Supporting Cells but Not in Hair Cells

In the mammalian auditory system, work has shown that extracellular ATP acting on P2RY1 receptors triggers ER calcium release; this pathway is required for spontaneous calcium activity in supporting cells (Babola et al., 2020; Babola et al., 2021). Therefore, we used pharmacology to investigate whether P2RY1 signaling is required for initiating spontaneous calcium activity in supporting cells within the zebrafish lateral-line system.

For our analyses we measured cytoGCaMP6s signals in supporting cells in developing neuromasts. Using this approach, we found that the application of P2RY1 receptor antagonist MRS2500 significantly reduced the magnitude and frequency of spontaneous cytoGCaMP6s signals in zebrafish supporting cells (example, day 3, Figures 2A1–A2; Supplementary Figures S3A–A′). This result indicates that P2yr1 is required for spontaneous activity in zebrafish supporting cells. To determine if ER calcium was released downstream of P2ry1 in zebrafish supporting cells, we examined spontaneous calcium activity before and after inhibitors of ER calcium release. For our analysis, we used thapsigargin to block the Sarco/ER calcium ATPase (SERCA). SERCA block prevents reentry of calcium to the ER from the cytosol and ultimately depletes ER calcium stores (Courjaret et al., 2018). After application of thapsigargin, we observed that the baseline cytoGCaMP6s levels increased dramatically in supporting cells within 5 min (example, day 3, Figures 2C1–C1′). In addition to changes in baseline calcium, we also observed that the application of thapsigargin dramatically reduced spontaneous calcium activity in supporting cells (example, day 3, Figure 2C2).

To further support the idea that ER release from the cytosol acts downstream of P2yr1 receptors, we examined the spatiotemporal properties of spontaneous calcium activity in supporting cells more closely. For our analysis, we imaged supporting cells in cross-section longitudinally. We acquired cytoGCaMP6s signals in a single plane, at a faster image acquisition speed (10 fr/s; in Figure 2 each volume was acquired in 200 ms every 3 s). We used heat maps to illustrate the spatial increase in calcium signals during a representative spontaneous event (Supplementary Figure S4A1). We also plotted the calcium signals at three distinct positions within the cell (Supplementary Figures S4A2–A4: top, middle, and bottom). Both the heat maps and plots revealed that spontaneous calcium activity in supporting cells initiates in the upper regions of the cell. These plots revealed that within 0.5 s after initiation, a calcium increase was observed throughout the entire supporting cell, with activity at the top of the cell detected ∼300 ms before activity at the bottom of the cell. This apical initiation is interesting because it is the proposed location of the purinergic receptors present in supporting cells within the mammalian auditory system (Babola et al., 2021). In this scenario, the delay in calcium signal at the base of the supporting cell could be due to diffusion of calcium released from the ER at the apex of the cell.

Lastly, we also tested whether P2yr1 or SERCA block altered spontaneous calcium activity in developing hair cells. Using cytoGCaMP6s, we found that P2yr1 block with MRS2500 did not impact spontaneous calcium activity in immature hair cells (example, day 3, Figures 2B1–B2; Supplementary Figures S3B–B′). Similarly, we examined the impact of SERCA block on spontaneous calcium activity in immature hair cells. We found that neither the baseline calcium (Figures 2D1–D1′) nor the spontaneous calcium signals (Figure 2D2) were significantly altered after thapsigargin application. These data indicate that neither P2yr1 receptor function nor ER calcium stores are critical for spontaneous calcium activity in immature hair cells.

Overall, we found that in the zebrafish lateral line, a P2yr1-ER signaling cascade is required for spontaneous calcium activity in supporting cells. While the pharmacological block of P2yr1 or ER calcium stores dramatically blocks spontaneous calcium activity in supporting cells, this same block does not alter spontaneous calcium activity in zebrafish hair cells. These pharmacological results indicate that during development, zebrafish hair cells and supporting cells use distinct mechanisms to generate spontaneous calcium signals. In addition, these results provide further evidence that in the zebrafish lateral line, spontaneous calcium activity in hair cells does not require concomitant activity in surrounding supporting cells.

### Two-Color Imaging Reveals No Link Between Spontaneous Activities in Hair Cells and Efferents

If supporting cells do not trigger spontaneous calcium activity in hair cells in zebrafish, then how is this activity generated in developing hair cells? In the developing zebrafish lateral line, cholinergic efferents synapse directly onto immature hair cells (Figure 3A1). But whether these efferents are spontaneously active or whether they regulate the spontaneous calcium activity in immature zebrafish lateral-line hair cells is not known.

To measure calcium signals in cholinergic efferents, we used a transgenic line expressing green cytoGCaMP6s (*UAS:cytoGCaMP6s* driven by *chat:Gal4*.) used this transgenic line in combination with a transgenic line expressing red cytoRGECO1 in hair cells (Figure 3A1) to monitor calcium activities in these two cell types simultaneously (Supplementary Movie S5). Using this approach, we found that in developing neuromast organs, without any external stimuli, the efferent terminals contacting hair cells were spontaneously active (examples, day 3, Figures 3A1–A2′; Supplementary Figures S6A1–A3′). In addition, we observed that among all efferent terminals within a neuromast, spontaneous calcium activities were highly synchronized (Supplementary Figure S6B, Pearson’s *R* = 0.92 ± 0.007, *n* = 4 neuromasts). While the magnitude and frequency of spontaneous calcium activity in efferents terminals was robust during development (day 3), upon lateral-line maturation (day 6) the activity was reduced (Supplementary Figures S6C–C′). Because spontaneous calcium activities in both efferent terminals and hair cells were largely restricted to development, we reasoned that these activities could be linked. But when we compared the spontaneous calcium activity in each efferent terminal with its hair cell target, we found no correlation (Figure 3B; Pearson’s *R* = 0.03 ± 0.02, *n* = 34 hair cell-efferent terminal pairs). This lack of correlation indicates that cholinergic efferent neurons may not directly trigger spontaneous calcium activity in hair cells.

To further examine the role of cholinergic efferents in hair cell spontaneous activity, we took a pharmacological approach. Cholinergic efferents share a molecular mechanism that is conserved among vertebrate hair cell systems. Acetylcholine released from these efferents acts on α9 or α10 nicotinic acetylcholine receptors (nAChR), which allows calcium to enter near the hair cell presynapse. This calcium influx activates calcium-dependent potassium SK channels leading to hyperpolarization of the hair cell. To determine if this cascade of events impacts hair cell spontaneous activity, we applied α-bungarotoxin (α-Btx) and apamin, compounds that block α9 nAChRs and SK channels respectively. Recent work has shown that these compounds are effective at disrupting efferent neurotransmission onto lateral-line hair cells (Carpaneto Freixas et al., 2021). Because cholinergic signaling is thought to impact the hair cell presynapse, for our analyses we used a membrane-localized indicator memGCaMP6s which we have used previously to specifically measure spontaneous presynaptic calcium activity (Hiu-Tung et al., 2019). We found that neither α-Btx nor apamin impacted spontaneous presynaptic calcium activity in immature hair cells (day 3, Figures 3C–C′). Overall, our imaging revealed that both hair cells and efferents have spontaneous calcium activities during development. Further, our two-color imaging and pharmacological results support the conclusion that cholinergic efferents are not required for spontaneous calcium activity in hair cells.

### Spontaneous Calcium Activity Occurs in the Mechanosensory Bundle and at the Presynapse

**FIGURE 4:**
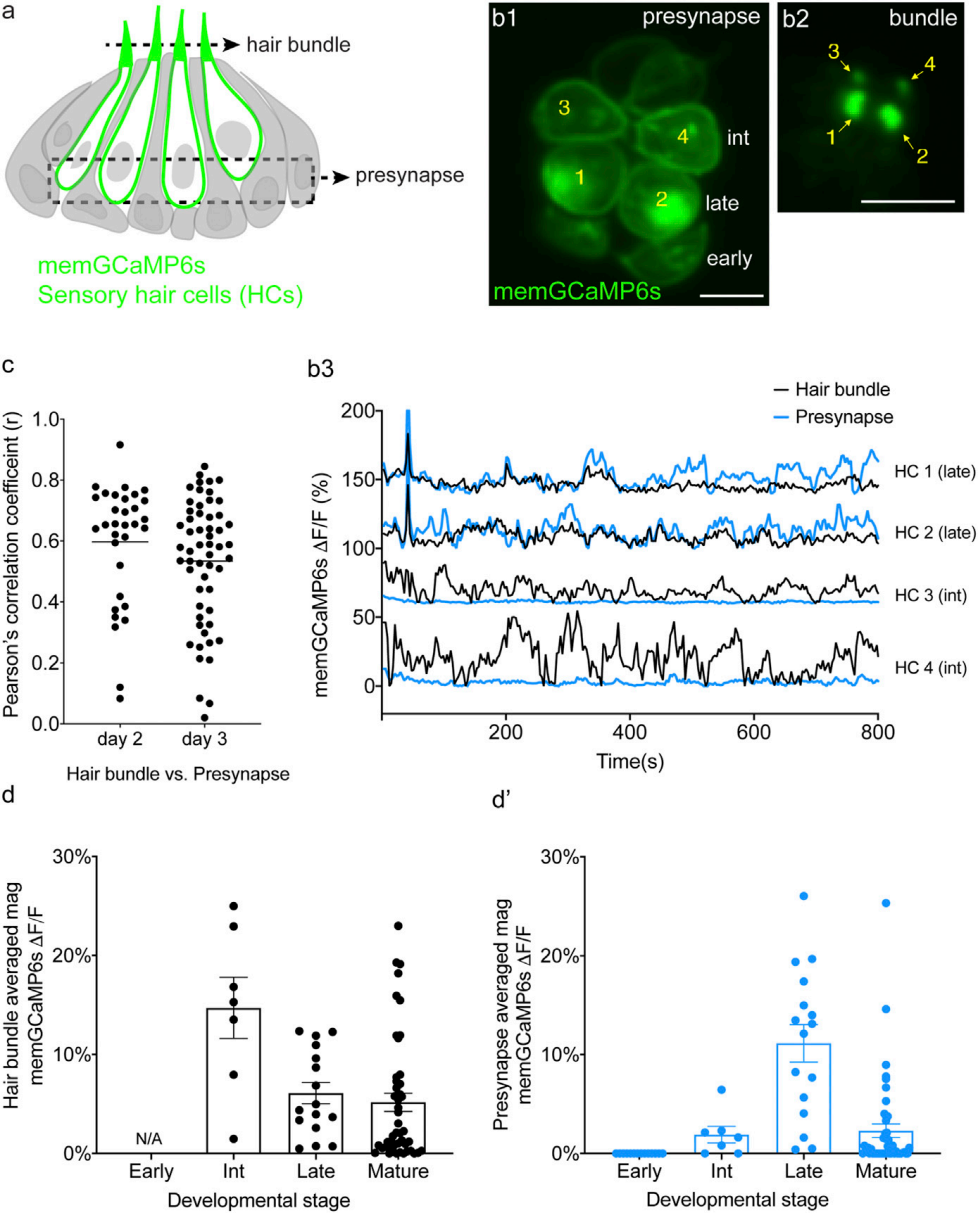
Spontaneous calcium activity occurs in the mechanosensory bundle and at the presynapse. **(A)** Cartoon of a neuromast organ illustrating hair cells (green) expressing memGCaMP6s, surrounded by supporting cells (gray). **(B1)** Image of a representative neuromast showing the hair cell presynaptic region indicated by the dashed box in **(A)** taken from immature hair cells at day 2. Hair cells at three different developmental stages are labeled: early (no detectable hair bundle), intermediate (3, 4), and late (1, 2). **(B2)** An image of the hair bundles from the same cells as **(B1)** through the plane indicated by the dash line in **(A)**. **(B3)** Paired temporal curves of spontaneous calcium activity in hair bundles (**B2** black) and the presynaptic regions (**B1** blue) taken from the same hair cells. **(C)** Pearson’s *R* values of spontaneous calcium activity between hair bundles and the presynaptic regions of the same sets of immature hair cells, *n* = 32 hair cells (*n* = 6 neuromasts), day 2 and *n* = 56 hair cells (*n* = 6 neuromasts), day 3. **(D,E)** The average magnitude of spontaneous calcium activity in hair bundles **(D)** and presynaptic region **(E)** in the same set of hair cells during the progressive stages of development: early, intermediate, late and mature. No activity can be measured in early hair bundles (N/A) as the bundles are not yet detectable. The spontaneous calcium activity in hair bundles peaks at the intermediate stage **(D)** and decreases upon maturation, while spontaneous calcium activity in the presynaptic regions peaks at the late stage **(D′)** and decreases upon maturation. Scale bar = 5 μm.

Our two-color calcium imaging (Figures 1A–C, 3A1′–A3′) indicates that in the zebrafish lateral line, neither supporting cells nor cholinergic efferent neurons–two cell types that are spontaneously active and directly contact hair cells–trigger hair cell spontaneous calcium activity. Therefore, we hypothesized that spontaneous calcium activity in hair cells may be generated autonomously. In mature hair cells the main pathway leading to calcium influx are generated in response to sensory stimuli. In response to stimuli, mechanoelectrical transduction (MET) channels open, leading to a cationic influx in the mechanosensory hair bundle that can trigger opening of presynaptic calcium channels at the presynapse (Pickles et al., 1984). (Figure 4A). Therefore, one possibility is that spontaneous MET channel activity also triggers opening of presynaptic calcium channels during development.

To measure spontaneous calcium activity in mechanosensory bundles, we used a membrane-localized GECI memGCaMP6s. Previously we have used memGCaMP6s to measure evoked calcium signals in the mechanosensory bundle as well as both evoked and spontaneous calcium influx at the presynapse (Zhang et al., 2018; Hiu-Tung et al., 2019). Using memGCaMP6s, in the absence of mechanical stimuli, we observed robust, spontaneous calcium activity in apical mechanosensory bundles (Supplementary Movie S6). We examined spontaneous calcium activity in mechanosensory bundles in immature (day 3) and mature (day 6) hair cells. We found that similar to measurements using cytoGCaMP6s (Figures 1D–D′) and memGCaMP6s examining presynaptic activity (Hiu-Tung et al., 2019) (Supplementary Figures S8B–B′), spontaneous activity in mechanosensory bundles was largely restricted to immature hair cells (Supplementary Figures S8A–A′).

By acquiring top-down views of neuromasts, we were able to isolate and simultaneously measure activities in both a basal, presynaptic plane (example, Figures 4B1–B3) and an apical mechanosensory bundle plane (example, Figure 4B2) within the same hair cells. When we examined individual hair cells, in many cases, we found that the spontaneous calcium activity in each apical mechanosensory bundle and its respective presynaptic region were highly correlated (example, Figure 4B3, see HC 1 and HC 2). In other cases, we observed hair cells with spontaneous calcium activity in the mechanosensory bundle but not in the presynaptic region (example, Figure 4B3, see HC 3 and HC 4). On average, the correlation in spontaneous calcium activity at the apex and the base of individual hair cells was high in immature hair cells (Figure 4C, day 2, Pearson’s *R* = 0.60 ± 0.035, *n* = 32 hair cells; day 3 Pearson’s *R* = 0.54 ± 0.027, *n* = 56 hair cells). This relatively high correlation indicates that spontaneous calcium activity at the presynaptic region may be driven by or be related to spontaneous calcium activity in the mechanosensory bundle.

Although there was an overall high correlation between spontaneous calcium activity in these two regions, we observed two distinct spontaneous activity profiles: 1) cells with synchronized spontaneous activity in the mechanosensory bundle and at the presynapse and 2) cells with spontaneous activity in the mechanosensory bundle with no accompanying activity at the presynapse. We looked more closely at individual hair cells at different stages of development to understand why there were two distinct profiles. For our analysis we examined neuromasts at day 2 when there are hair cells at different developmental stages. Previous work has demonstrated that in the lateral line, hair cell stage can be estimated by measuring the height of the tallest structure in the mechanosensory bundle, the kinocilium (Stage- hair bundle height: early- not detectable; intermediate- 1–10 μm; late- > 10 < 18 μm; mature- >18 µm. Lateral-line hair cells take roughly 20 h to complete this maturation) (Kindt et al., 2012; Dow et al., 2018). Overall, using this staging, we found that spontaneous calcium activity in both the mechanosensory bundle and the presynaptic compartment increased in strength during development and decreased upon hair cell maturation (Figures 4D–D′). In addition, we observed that spontaneous calcium activity in the mechanosensory bundle peaked at an earlier development stage compared to activity at the presynapse (Figures 4D–D′). Further, in younger hair cells, we observed spontaneous calcium activity in mechanosensory bundles but not in the presynaptic compartment (labeled as “int” in Figure 4B1; see HC 3 and HC 4 in Figures 4B1–B3). In slightly more mature hair cells, we observed spontaneous calcium activity in both the mechanosensory bundle and the presynapse (labeled as “late” in Figure 4B1; see HC 1 and HC2 in Figures 4B1–B3). At this relatively more mature stage, activity in the mechanosensory bundle and at the presynapse were highly correlated (see HC 1 and HC2 in Figures 4B1–B3). Thus, a differential onset in spontaneous activity (mechanosensory bundle preceding the presynapse) resulted in two distinct spontaneous activity profiles.

Overall, by measuring calcium activities at the membrane, we demonstrate for the first time that spontaneous calcium activity is present in both apical mechanosensory bundles and basal presynaptic compartments *in vivo*. At early stages of hair cell development, spontaneous calcium activity is present in mechanosensory bundles but not at the presynapse. At late stages of hair cell development spontaneous activity is present in both the mechanosensory bundle and the presynapse, and these activities are highly correlated.

### Ca_V_1.3 Channels are Required for Spontaneous Presynaptic- But Not Hair Bundle-Activity

Our memGCaMP6s-based calcium imaging indicates that spontaneous activity in immature hair cells occurs in two distinct subcellular compartments. But how these signals are initiated in lateral-line hair cells remained unclear. In the zebrafish lateral line, Ca_V_1.3, a L-type calcium channel is required for calcium influx at the hair cell presynapse (Hiu-Tung et al., 2019). Therefore, we used both genetics and pharmacology to examine whether Ca_V_1.3 channels are required for spontaneous calcium activity in hair cells of the lateral line.

**FIGURE 5:**
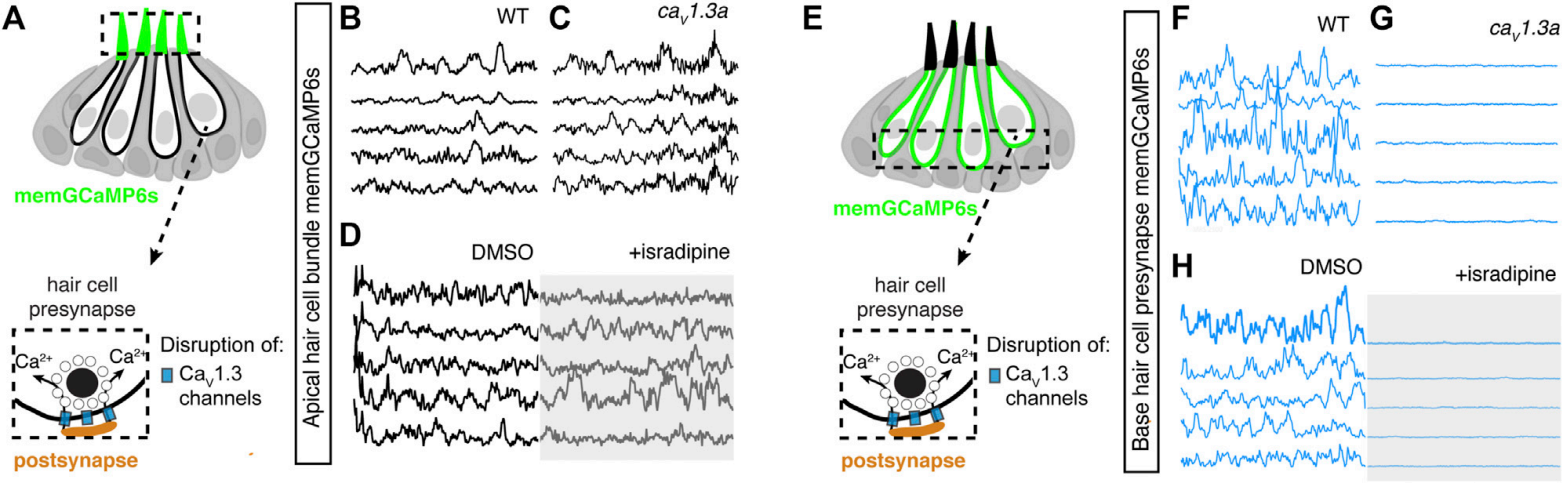
Spontaneous calcium activity at the presynapse requires Ca_V_1.3 channels. **(A)** Diagram illustrating the hair cells with the hair bundles highlighted by a dashed box where we measure their spontaneous calcium activity. The structure of a single ribbon synapse at the base of a hair cell is illustrated, as well as the calcium channels beneath it. **(B–D)** Representative memGCaMP6s temporal traces show that disruption of Ca_V_1.3a channels using *cav1.3a* mutants **(B,C)** or pharmacological block using 10 µM isradipine **(D)** does not impact spontaneous activity in apical hair bundles. **(E)** Diagram illustrating the hair cells with the presynaptic area highlighted by a dashed box where we measure the spontaneous presynaptic calcium activity. **(F–H)** Representative memGCaMP6s temporal traces show that disruption of calcium channels using *cav1.3a* mutants **(F–G)** or pharmacological block using 10 µM isradipine **(H)** completely blocked spontaneous activity at the hair cell presynapse. All measurements were performed in immature hair cells at day 3.

For our analyses, we examined activity in *ca*_*V*_ *1.3a* zebrafish mutants and in wildtype animals after treatment with isradipine, a Ca_V_1.3 channel antagonist. Both of these manipulations have been shown to block evoked presynaptic calcium influx in zebrafish hair cells (Sheets et al., 2012; Zhang et al., 2018). To measure spontaneous calcium activity, we used memGCaMP6s to detect signals in the mechanosensory bundle and at the presynapse. We found that in *ca*_*V*_ *1.3a* mutants or after acute block of Ca_V_1.3 channels with isradipine, spontaneous calcium activity in the mechanosensory bundle was not significantly changed compared to controls (Figures 5A–D; Supplementary Figures S9A–B′). However, we found that spontaneous calcium activity occurring at the hair cell presynapse was abolished in *ca*_*v*_ *1.3a* mutants (Figures 5E–G; Supplementary Figures S9C–D′) and after isradipine treatment (Figure 5H; Supplementary Figures S9C–D′). Together these manipulations indicate that spontaneous calcium activity can occur in the mechanosensory bundle without accompanying activity at the presynapse. Furthermore, they demonstrate that while Ca_v_1.3 channels are not required for spontaneous calcium activity in mechanosensory bundles, they are essential for spontaneous calcium activity at the presynapse.

### Mechanotransduction in Hair Bundles Is Required for Spontaneous Activity at the Presynapse

Our functional calcium imaging indicates that spontaneous calcium activity at the presynapse, when present, occurs concomitantly with activity in the mechanosensory bundle (Figure 4C). Additionally, in mature hair cells, evoked activity opens MET channels leading to an influx of cations, including calcium, that triggers opening of Ca_V_1.3 channels at the presynapse (Bechstedt and Howard, 2007). Therefore, we hypothesized that the spontaneous opening of MET channels in mechanosensory bundles might trigger activity at the presynapse. To test this hypothesis, we used genetics and pharmacology to determine whether MET channel function is required to trigger spontaneous calcium activity at the presynapse of immature hair cells.

**FIGURE 6:**
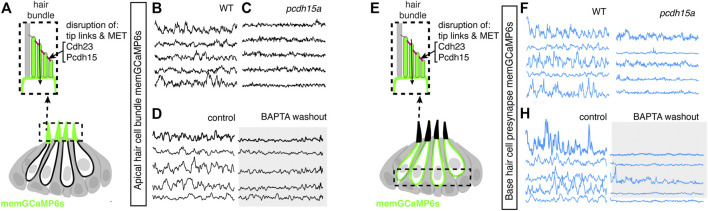
Mechanotransduction in hair bundles is required for spontaneous activity at the presynapse. **(A)** Diagram illustrating the hair cells with the hair bundles highlighted by a dashed box where we measure their spontaneous calcium activity. The structure of the hair bundle from a single hair cell at is illustrated on top. The mechanotransduction (MET) channels are gated by tip links made up of Pcdh15a Cdh23. Pharmacological application of BAPTA, an extracellular calcium chelator, breaks tip links and acutely disrupts MET function. **(B–D)** Representative GCaMP6s temporal traces show that disruption of tip links using *pcdh15a* mutants **(B,C)** or by pharmacologically disrupting tip links using BAPTA **(D)** disrupts spontaneous activity in hair bundles. **(E)** Diagram illustrating the hair cells with the hair cell presynaptic area highlighted by a dashed box at the base of the hair cells where we measure the spontaneous presynaptic calcium activity. **(E–H)** Representative GCaMP6s temporal traces show that disruption of tip links using *pcdh15a* mutants **(F–G)** or by pharmacologically disrupting tip links using BAPTA **(H)** also disrupts spontaneous calcium activity at the presynapse. All measurements were performed in immature hair cells at day 3.

For our genetic analysis, we used the zebrafish mutants lacking Protocadherin 15a (PCDH15), a core component of hair cell tip-link, a structure required to gate MET channels in mature hair cells (Seiler et al., 2005) (Figure 6A). For our pharmacological analysis, we applied BAPTA, an extracellular calcium chelator that breaks tip links and acutely disrupts MET channel function. We found that MET channel disruption in *pcdh15a* mutants or after BAPTA treatment significantly reduced spontaneous calcium activity in mechanosensory bundles (Figures 6A–D; Supplementary Figures S10A–B′). This indicates that in the lateral line, both evoked calcium activity in mature mechanosensory bundles and spontaneous calcium activity in developing mechanosensory bundles depends on MET function. Importantly, we found MET channel disruption also dramatically blocked spontaneous calcium activity at the hair cell presynapse (Figures 6E–H; Supplementary Figures S9C–D′). After our manipulations we did observe some residual spontaneous calcium activity in the mechanosensory bundle and at the presynapse, which may reflect incomplete block of MET channel function. Overall, this result indicates that spontaneous activity at the presynapse is primarily driven by the spontaneous opening of MET channels. Thus, it is spontaneous MET that generates spontaneous calcium activity at the presynapse, in order to shape synapse formation and propagate information downstream to the developing sensory system.

## Discussion

**FIGURE 7:**
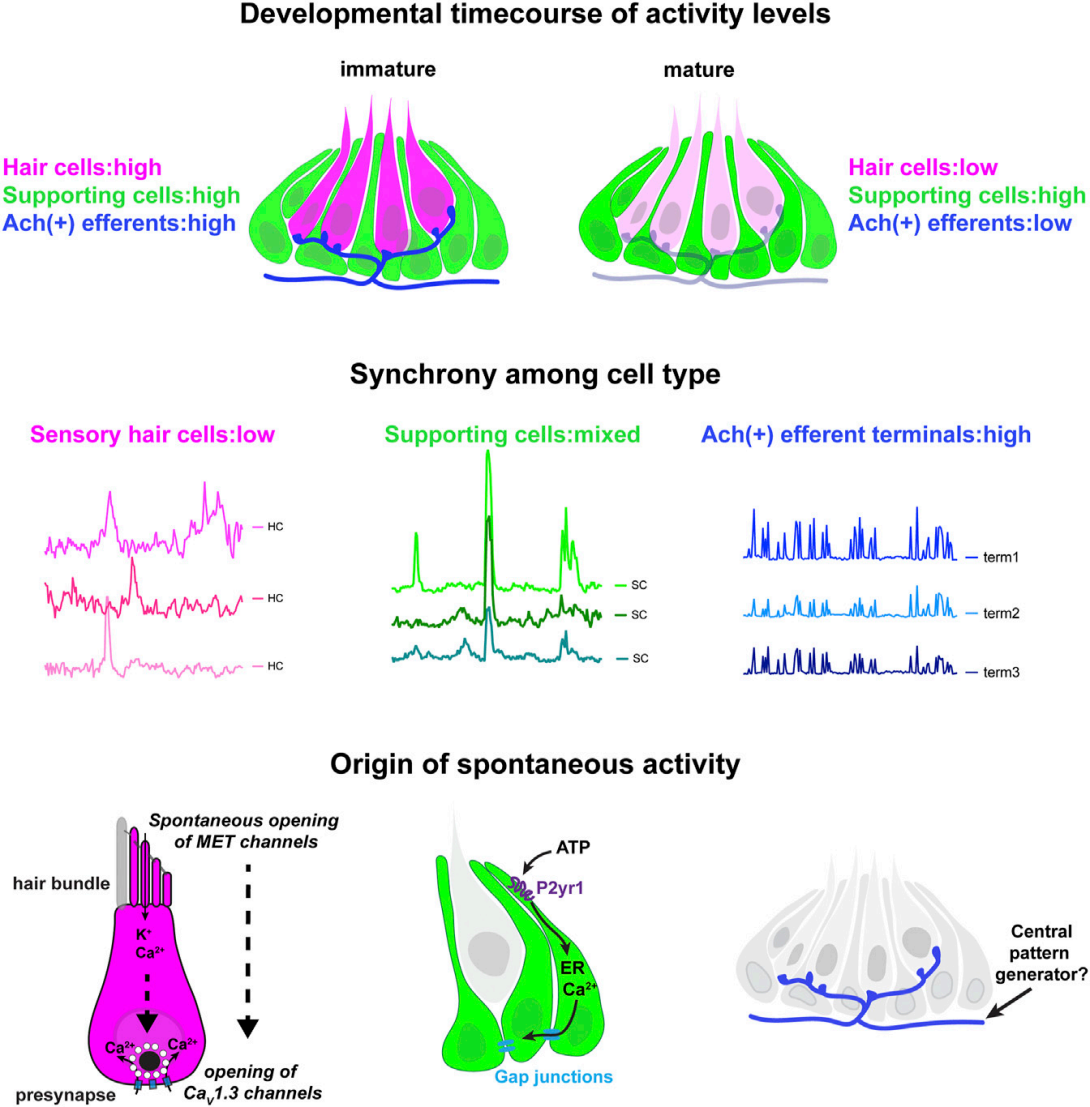
Locations, mechanisms and developmental timecourse of spontaneous calcium activities in the lateral-line system. Top panel: we reliably detected spontaneous calcium activity in three cell types within the lateral-line system: hair cells, supporting cells and cholinergic (Ach (+)) efferent neurons. Spontaneous calcium activity in the supporting cells and cholinergic efferents is not correlated with calcium signals in hair cells. During development spontaneous calcium signals are robust in all three cell types (left side). Upon maturation, spontaneous calcium signals are decreased in hair cells and efferent neurons, but maintained in supporting cells (right side). Middle panel: Within a neuromast spontaneous calcium signals among populations of hair cells are not correlated (left side); supporting cells are moderately correlated (middle); cholinergic terminals are highly correlated (right side). Bottom panel: In developing hair cells, spontaneous opening of MET channels leads to calcium signals in mechanosensory hair bundles. This apical activity triggers opening of Ca_V_1.3 channels at the presynapse, resulting in spontaneous presynaptic calcium signals (left side). In supporting cells, extracellular ATP acts on P2yr1 receptors. P2yr1 signaling leads to calcium release from the ER, giving rise to a spontaneous calcium signal. Spontaneous calcium signals can be propagated to neighboring supporting cells via gap junction channels (middle). Spontaneous activity in cholinergic efferents may be coupled to activity in spinal motor neurons that is present during development in order to form the central pattern generator required for locomotion (right side).

In this work, we show that zebrafish can be used as an *in vivo* model to study spontaneous activity in developing hair cell sensory systems. The origin and role of spontaneous activity in hair cell systems is an important area of research in sensory neuroscience. Our work uses genetics, pharmacology, GECIs along with cutting-edge LSFM to image and characterize spontaneous calcium activity in the lateral-line system. We find that spontaneous activity is present in hair cells, supporting cells and efferent terminals*–*spontaneous activity in each cell type occurs with largely distinct developmental, temporal and mechanistic origins (Figure 7). Overall, our work demonstrates that the lateral line presents a robust model to study many facets of spontaneous activity, all within an intact sensory system.

### The Origin of Spontaneous Calcium Activity in the Zebrafish Hair Cells

In our study on the zebrafish lateral line, we find that spontaneous calcium signals occur autonomously in hair cells. In support of this finding, we show that hair cell spontaneous activity is still intact when supporting cell spontaneous activity is blocked (Block P2ry1 receptors or ER calcium release, Figure 2; Supplementary Figure S3) or when the cholinergic receptors on hair cells are blocked (Block α9 nAChRs, Figures 3C–C′). This indicates, that in the zebrafish lateral line, neither supporting cells nor efferent neurons have a striking impact on spontaneous calcium activity in developing hair cells.

Based on our work we hypothesize that the spontaneous calcium signals we observe in lateral-line hair cells are analogous to the uncoordinated calcium signals that have been observed in the developing auditory and vestibular systems of mice (Eckrich et al., 2018; Holman et al., 2019). In mice these calcium signals have been visualized using either cytosolic GECIs or calcium dyes, or by recording calcium action potentials using electrophysiology. Our work uses both a cytosolic GECI (cytoGCaMP6s) as well as a membrane-localized GECI (memGCaMP6s) to visualize spontaneous calcium signals in lateral-line hair cells (Figures 1, 4). Importantly, using memGCaMP6s, we find that spontaneous activity within hair cells occurs in two spatially distinct domains–the mechanosensory bundle and the presynapse (Figure 4). Using memGCaMP6s in lateral-line hair cells, we find that Ca_V_1.3 channels, present at the developing presynapse, are essential for spontaneous presynaptic calcium signals (Figures 5E–H; Supplementary Figures S9C–D′). Importantly, both work in mice and zebrafish indicates that during development, Ca_V_1.3-dependent, presynaptic calcium activity is critical for proper hair cell synapse formation.

Interesting, our work found that disrupting Ca_V_1.3 channel function did not alter spontaneous calcium activity in mechanosensory bundles. This result supports the idea that spontaneous calcium activity in mechanosensory bundles occurs upstream of activity at the presynapse. In support of this idea, we found that 1) activity in these two domains is highly correlated (Figures 4B3–C) 2) at the earliest stages of hair cell development spontaneous activity was present in mechanosensory bundles but not at the presynapse (Figures 4B3, D–D′) and 3) blocking MET channel function significantly reduced spontaneous calcium activity at the presynapse (Figures 6E–H; Supplementary Figures S10C–D′). Together our results show that in developing lateral-line hair cells, MET activity initiates autonomously generated spontaneous events. Further, this activity in hair bundles triggers presynaptic calcium influx. Work in mice has shown that MET activity in developing hair cells is important for hair cell survival, hair cell synapse maintenance, and for the proper maturation of hair cells, afferent neurons and efferent neurons (Marcotti et al., 2006; Corns et al., 2018; Sun et al., 2018). Therefore, during development, spontaneous MET function in the mechanosensory bundle likely plays many important roles.

### The Origin and Role of Spontaneous Calcium Activity in the Zebrafish Supporting Cells

Together, our study in zebrafish along with work in mammals demonstrates that spontaneous calcium signals in supporting cells are a conserved feature among hair cell systems (Figures 1B1–B1′, B4) (Tritsch et al., 2007; Holman et al., 2019). Unlike the mouse auditory system, in the lateral line, waves of spontaneous activity in supporting cells did not propagate across neuromast organs. Interestingly, we did observe that activity between neighboring supporting cells was coupled in the lateral line (Figures 1B4–C; Supplementary Figures S2D1,D3). The level of coupling was higher in the outer most layer of supporting cells (mantle cells) compared to the more central supporting cells (Supplementary Figures S5A–B). In the future it will be interesting to examine spontaneous activity in these different subsets of supporting cells to understand the role of this activity.

Our findings indicate that the molecular pathway required to trigger spontaneous calcium signals in supporting cells may also be conserved between mammals and zebrafish. Our work in zebrafish indicates a P2yr1 signaling cascade leads to calcium release from the ER; this calcium release gives rise to the spontaneous calcium signals in lateral-line supporting cells (Figures 2A1,A2,C1,C2). This is consistent with recent work on supporting cells in the mouse auditory epithelium (Babola et al., 2020). Further, studies have shown that application of ATP evokes calcium signals in supporting cells within the auditory and vestibular epithelium–these signals also require ER calcium release (Dulon et al., 1993; Tritsch et al., 2007; Holman et al., 2019). Together these studies point towards a conserved P2-ER calcium axis that drives calcium signals in the supporting cells of hair cell epithelia. In the future, by using GECIs along with ATP sensors (Lobas et al., 2019) and two-color imaging, the zebrafish lateral line presents a useful model to study the dynamics of calcium and ATP *in vivo* during spontaneous events.

Although the molecular cascade giving rise to calcium signals in supporting cells appears largely conserved among hair cell systems, we did observe one striking difference in the lateral-line system. While spontaneous activity in supporting cells is transient in the mouse auditory and vestibular systems, it remains robust and constant in zebrafish lateral-line organs, even after the system is mature (Figures 1E–E′). The retention of spontaneous activity in supporting cells in the zebrafish lateral line after maturation could also be related to another difference between hair cell systems in zebrafish (and other nonmammalian vertebrates) and mammals–the ability to regenerate hair cells (Corwin and Warchol, 1991; Thomas and Raible, 2019). For example, in zebrafish, after hair cell loss, supporting cells proliferate to give rise to new hair cells–a process that happens rarely in mature hair cell systems in mammals. The retention of spontaneous calcium signals in zebrafish supporting cells may be important to trigger gene expression required for cell proliferation during regeneration. In this scenario, the retention of spontaneous activity in supporting cells could reflect a feature of their innate regenerative capacity.

### The Origin and Role of Spontaneous Calcium Activity in Zebrafish Cholinergic Efferents

Our work demonstrates that cholinergic efferents in the lateral-line system are spontaneously active (Figures 3A1–A2′; Supplementary Figures S6A1–A2′). In addition, spontaneous activity in cholinergic efferents is stronger during development, indicating it may play a vital developmental role (Supplementary Figures S6C–C′). Numerous studies have investigated the role of cholinergic efferents in mature hair cell systems. In this context, cholinergic efferents are inhibitory, either through contacts with hair cells (outer auditory hair cells, type II vestibular hair cells, lateral-line hair cells) or afferents terminals (inner auditory hair cells, type I vestibular hair cells) (Housley and Ashmore, 1991; Lunsford et al., 2019; Poppi et al., 2020). But what role these efferents play during development is less clear. Our study suggests the activity of cholinergic efferents does not directly influence or trigger spontaneous calcium activity in immature lateral-line hair cells (Figures 3C–C′). It is possible that under the native conditions present within our system, the amount of acetylcholine released from the efferent fibers is not sufficient to alter hair cell calcium to a level detectable using GECIs. It is also possible that acetylcholine may act on supporting cells, other efferent neurons, or afferents neurons in the developing lateral line. In the future it will be important examine the impact and target of cholinergic efferent neurons in the lateral line more closely, in the context of development.

In addition to studies focused on the periphery, work in mice has shown that hair cell spontaneous activity is mirrored downstream, in the central auditory pathway. Furthermore, spontaneous activity from each ear is patterned centrally, in a bilateral manner. Recent work has shown that cholinergic efferent neurons are required to ensure that spontaneous activity is patterned in a bilateral manner (Babola et al., 2021). In our work, we demonstrated that spontaneous activity is highly correlated at all efferent terminals within a neuromast. We also found that correlated activity in the cholinergic efferent (likely the same fiber) extends to multiple neuromasts along one side of the fish body (Supplementary Figures S7A,B). Therefore, it is possible that cholinergic efferents may regulate hair cell or afferent spontaneous activity in a manner that is bilateral (left versus right side of the fish body). In the future it will be interesting to develop methods to image activity from cholinergic efferents innervating each side of the zebrafish body. In addition, it will be interesting to determine how hair cell spontaneous activity is reflected in the brain, and whether cholinergic efferents impact the patterning of this activity more centrally.

Could cholinergic efferents pattern bilaterality? Recent work has shown in mature zebrafish larvae, during swimming cholinergic efferents are synchronously active with spinal motor neurons (Lunsford et al., 2019). Therefore, one possibility is that during development, spontaneous activity in cholinergic efferents may also reflect the activity of spinal motor neurons. During spinal cord development, the central pattern generator (CPG) is established, in order to create the oscillatory rhythms needed for locomotion (Grillner et al., 1998). As part of CPG maturation, a rhythmic alternation between the two sides of the spinal cord is established. This motor prerequisite to swimming behavior may also synchronize with spontaneous activity in cholinergic efferents of the lateral line. If the cholinergic efferents set up bilateral patterning of the lateral line, tapping into the activity of the developing spinal motor neurons and the CPG is a viable way for this to occur (Figure 7).

In summary our work provides a comprehensive characterization of spontaneous activity in the zebrafish lateral line. Using LSFM we demonstrate that in neuromast organs, spontaneous activity is present in hair cells, supporting cells and cholinergic efferent terminals. Within the lateral-line, each of these activities may play unique roles in sensory system function, development, maintenance, regeneration, and response to damage. Exploring the role of these spontaneous activities in these important biological contexts will make for exciting future work.

## Data Availability

The raw data supporting the conclusion of this article will be made available by the authors, without undue reservation. Matlab R2020a used to process functional imaging data and the code is available upon request.
